# A new species of the genus *Sweltsa* Ricker, 1943 (Plecoptera, Chloroperlidae) from Guizhou Province, China

**DOI:** 10.3897/BDJ.10.e80433

**Published:** 2022-04-15

**Authors:** Abdur Rehman, Qing-Bo Huo, Yu-Zhou Du

**Affiliations:** 1 School of Horticulture and Plant Protection & Institute of Applied Entomology, Yangzhou University, Yangzhou 225009, China School of Horticulture and Plant Protection & Institute of Applied Entomology, Yangzhou University Yangzhou 225009 China; 2 Joint International Research Laboratory of Agriculture and Agri-Product Safety, the Ministry of Education, Yangzhou University, Yangzhou 225009, China Joint International Research Laboratory of Agriculture and Agri-Product Safety, the Ministry of Education, Yangzhou University Yangzhou 225009 China

**Keywords:** China, Chloroperlidae, Guizhou Province, new species, *Sweltsaligula* sp. n.

## Abstract

**Background:**

*Sweltsa* is a genus of green stoneflies in the family Chloroperlidae and is distributed throughout the Nearctic and East Palaearctic Regions. As they are sensitive to pollutants, they are often used as an indicator species for determining the quality of water bodies. There are around 57 species of this genus worldwide and 11 of those have been identified from China.

**New information:**

A new species of the Alloperlini genus *Sweltsa* Ricker, 1943, *Sweltsaligula* Rehman, Huo & Du sp. n. is described from Kuankuoshui National Natural Reserve, Suiyang County, Guizhou Province, southwest China. This is the first report of the family Chloroperlidae from Guizhou Province. Diagnosis, description of male, female and nymph, illustration of terminalia and similarities with closely-related species are provided and discussed.

## Introduction

The family Chloroperlidae Okamoto, 1912, a member of the superfamily Perloidea, contains only two subfamilies: Chloroperlinae Okamoto, 1912 and Paraperlinae Ricker, 1943. In China, six genera of Chloroperlidae are presently recorded: *Alloperla* Banks, 1906, *Alaskaperla* Stewart & DeWalt 1991, *Haploperla* Navás, 1934, *Suwallia* Ricker, 1943, *Utaperla* Ricker, 1952 and *Sweltsa* Ricker, 1943 (Wu 1938, Nelson and Hanson 1968, Nelson and Hanson 1969, Zhiltzova and Zwick 1971, Wu 1973, Du 1999, Stark and Sivec 2009, Li and Wang 2011, Li et al. 2013, Li et al. 2014, Li et al. 2015a, Li et al. 2015b, Chen and Du 2015, Chen and Du 2016a, Chen and Du 2016b, Yang and Li 2018, Chen 2019, Li et al. 2021a, Li et al. 2021b,Shi et al. 2022, Rehman et al. 2022).

The genus *Sweltsa* Ricker, 1943 belongs to the tribe Alloperlini of subfamily Chloroperlinae, with 57 species known worldwide (Teslenko and Zhiltzova 2009, Li et al. 2021a, DeWalt et al. 2022). *Sweltsa* was proposed by Ricker (1943) as a subgenus of *Alloperla* and given generic status by Illies (1966). Presently, 11 species of this genus are known from China (Wu 1938, De Figueroa and Fochetti 2002, Li et al. 2014, Li et al. 2017, Chen and Du 2017, Dong et al. 2018, Mo et al. 2020, Li et al. 2021a). This study represents the first record of the family Chloroperlidae from Guizhou Province, China. Guizhou is rich in species diversity and lies in southwest China, borders the autonomous region of Guangxi to the south, Yunnan to the west, Sichuan to the northwest, the Municipality of Chongqing to the north and Hunan to the east. In this paper, the detailed descriptions, illustrations of male, female and nymph of the new species are provided and discussed.

## Materials and methods

The specimens were collected by aerial net or by hand and preserved in 75% ethanol. Terminalia were examined and illustrated by KEYENCE VHX-5000 and the final images were prepared using Adobe Photoshop CS6. The specimens were placed in the Insect Collection of Yangzhou University (ICYZU), Jiangsu Province, China. The morphological terminology of Surdick (1985), Stark and Sivec (2009) and Li et al. (2021a) were followed.

## Taxon treatments

### Sweltsa ligula

Rehman, Huo & Du sp. n.

0EE14131-FF03-5450-94FB-1794D6F08C6A

95A4A52C-B1A5-44A7-BB3C-3D98BEC3BA07

#### Materials

- **Type status:** Holotype. **Occurrence:** recordedBy: Du Yu-Zhou, Huo Qing-Bo; Yuan Jia-wen; individualID: Insect collection of Yangzhou University (ICYZU), Jiangsu Province, China; individualCount: 1; sex: 1 males; lifeStage: Adult; **Taxon:** scientificName: *Sweltsaligula*; kingdom: Animalia; phylum: Arthropoda; class: Insecta; order: Plecoptera; family: Chloroperlidae; genus: Sweltsa; specificEpithet: *ligula*; taxonRank: Species; **Location:** continent: Asia; country: China; countryCode: CN; stateProvince: Guizhou; county: Suiyang County; locality: Kuankuoshui National Natural Reserve; verbatimElevation: 1435; verbatimLatitude: 28°13.205′N; verbatimLongitude: 107°9.95′E,; **Identification:** identifiedBy: Rehman, Huo, Du; **Event:** year: 2019; month: 5; day: 5; **Record Level:** language: en; basisOfRecord: PreservedSpecimen.
- **Type status:** Paratype. **Occurrence:** recordedBy: Du Yu-Zhou, Huo Qing-Bo; Yuan Jia-wen; individualID: Insect collection of Yangzhou University (ICYZU), Jiangsu Province, China; individualCount: 7; sex: 1 males, 2 femles; lifeStage: 3 adults, 4 nymphs; **Taxon:** scientificName: *Sweltsaligula*; kingdom: Animalia; phylum: Arthropoda; class: Insecta; order: Plecoptera; family: Chloroperlidae; genus: Sweltsa; specificEpithet: *ligula*; taxonRank: Species; **Location:** continent: Asia; country: China; countryCode: CN; stateProvince: Guizhou; county: Suiyang County; locality: Kuankuoshui National Natural Reserve; verbatimElevation: 1435; verbatimLatitude: 28°13.205′N; verbatimLongitude: 107°9.95′E,; **Identification:** identifiedBy: Rehman, Huo, Du; **Event:** year: 2019; month: 5; day: 5; **Record Level:** language: en; basisOfRecord: PreservedSpecimen.

#### Description

**Figure 1.**
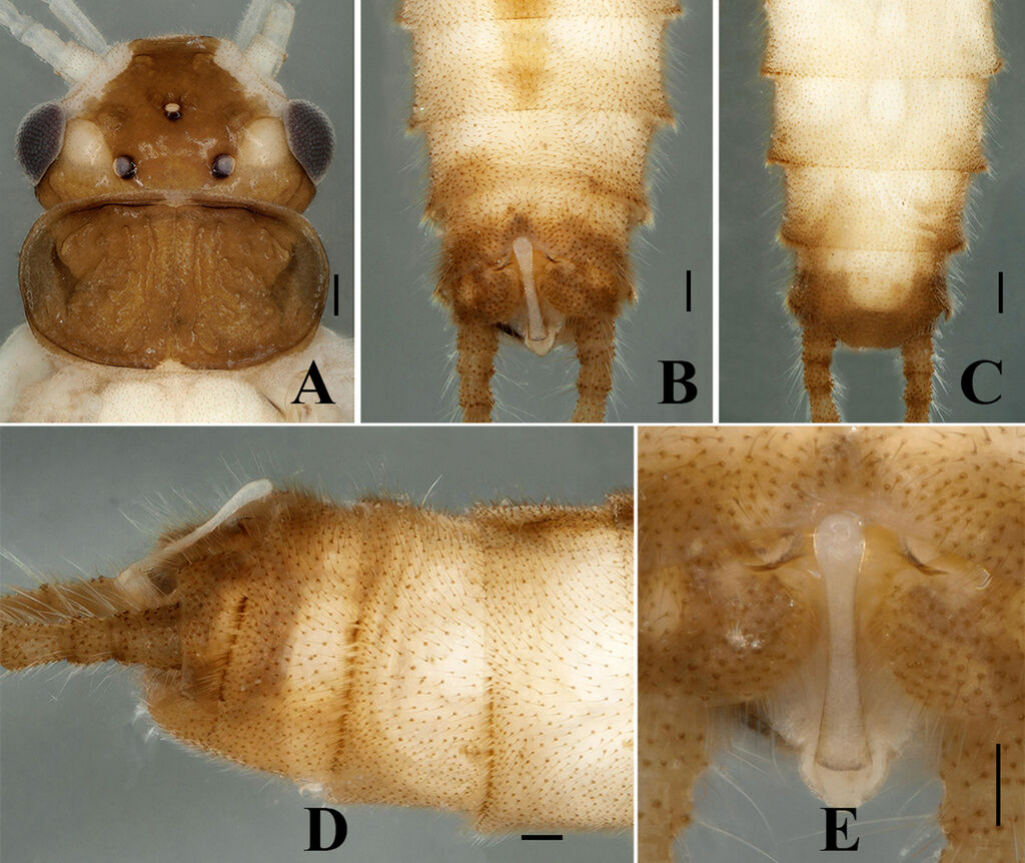
*Sweltsaligula* Rehman, Huo & Du sp. n. male holotype. **A** head and pronotum dorsal view; **B** terminalia dorsal view; **C** terminalia ventral view; **D** terminalia lateral view; **E** epiproct. Scale bar 0.1 mm.

**Figure 2.**
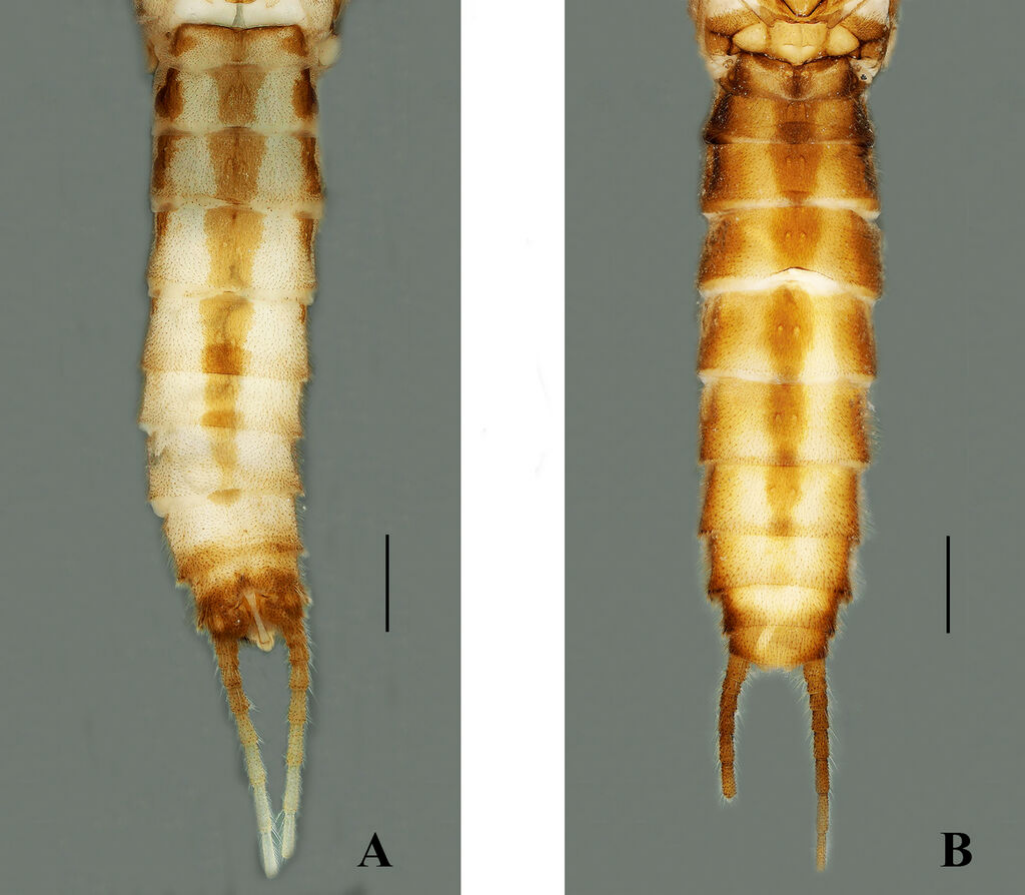
*Sweltsaligula* Rehman, Huo & Du sp. n. **A** male holotype, abdomen dorsal view; **B** female paratype, abdomen dorsal view. Scale bar 1 mm.

**Adult habitus.** Triocellate. General colour greenish in the field, but becoming pale brown in ethanol. The head is also black in the field, but changes to brown in ethanol. Head with rounded pale yellow spot between compound eye and lateral ocellus, frons dark brown from epicranial suture to clypeus and with broad pale areas along lateral margins adjacent to antennal bases. Compound eyes dark greyish, ocelli greyish, anterior ocellus paler with dark black margins; antennae and palpi pale. The pronotum disc is completely brown, bearing symmetrical rugosities, margin dark brown (Fig. 1A); mesonotae and metanotae brown with U-shaped marking, margin dark. Wings hyaline, forewing Rs branched, the anal field of hind-wing small and folded with three veins, legs pale. Abdominal terga 1–7 dorsally with brown median stripe of trapezoidal spots and tergum 8 with small rounded spot anteromedially, lateral dark patches also present on segments 1–4 (Fig. 2A). Cerci pale with long setae.

##### Male

**Figure 3.**
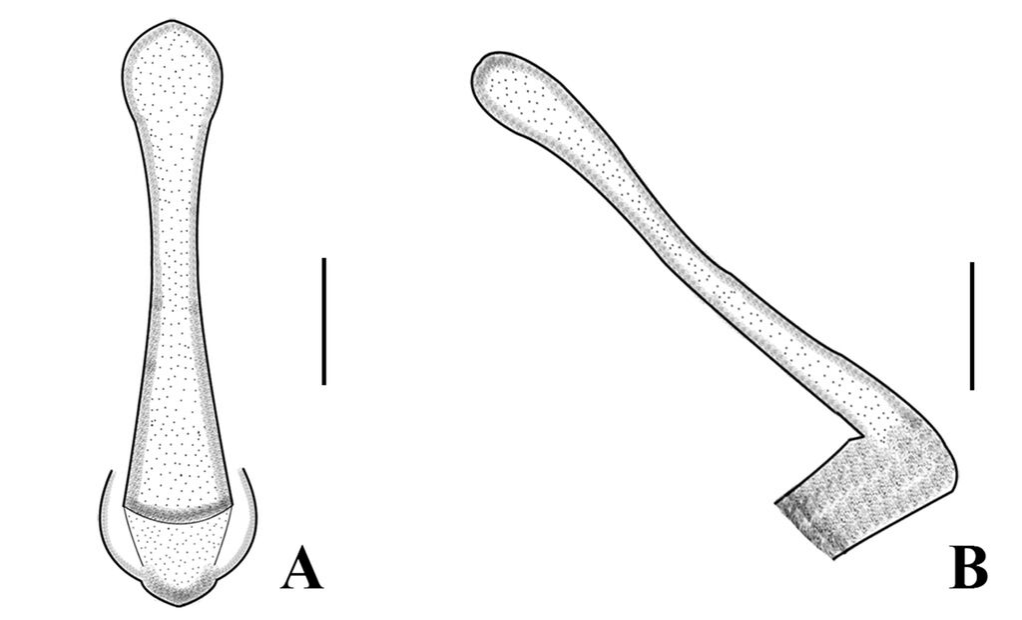
*Sweltsaligula* Rehman, Huo & Du sp. n. male holotype. **A** epiproct dorsal view; **B** epiproct lateral view. Scale bar 0.1 mm.

**Figure 4.**
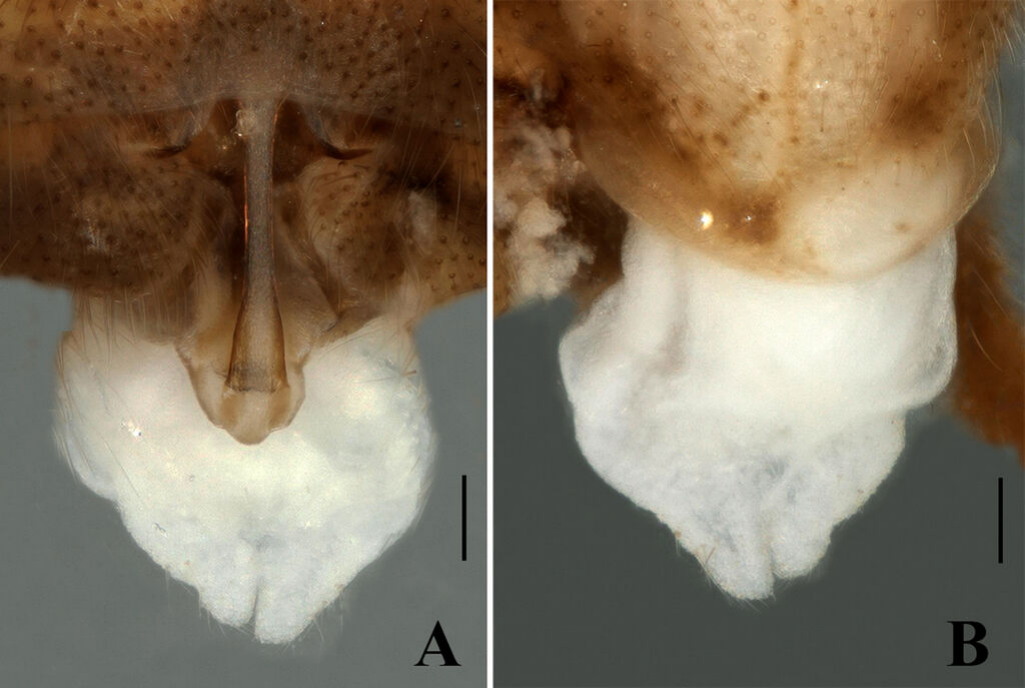
*Sweltsaligula* Rehman, Huo & Du sp. n. male paratype. **A** aedeagus dorsal view; **B** aedeagus ventral view. Scale bar 0.1 mm.

Body length 8.5–9.5 mm (n = 3); forewing length 7.0–7.5 mm, hind-wing length 6.2–6.8 mm. Tergum 9 sclerotised, posteriorly strongly sclerotised bearing long brown hairs, dorsally without any ridge and stripe. Tergum 10 divided medially; the medial portion enlarged, forming a shield-like structure with a dark brown basal anchor (Fig. 1B–D). Epiproct long and spoon-shaped, parallel-sided for most of its length, but constricted medially, broad from the basal portion, dorsolaterally sclerotised, apically rounded and apex forming spoon-bowl shape in dorsal view (Figs 1, 3A); in lateral aspect, the epiproct is thin and parallel for its most part, apically wide and slightly curved at the apex (Figs 1, 3B). Aedeagus is completely membranous, triangular in shape. The dorsal and dorsolateral lobes are slightly rounded in shape, basal portion bulbous. The posterior portion of the aedeagus bears a pair of finger-like apical lobes, each covered with numerous long hairs (Fig. 4).

##### Female

**Figure 5.**
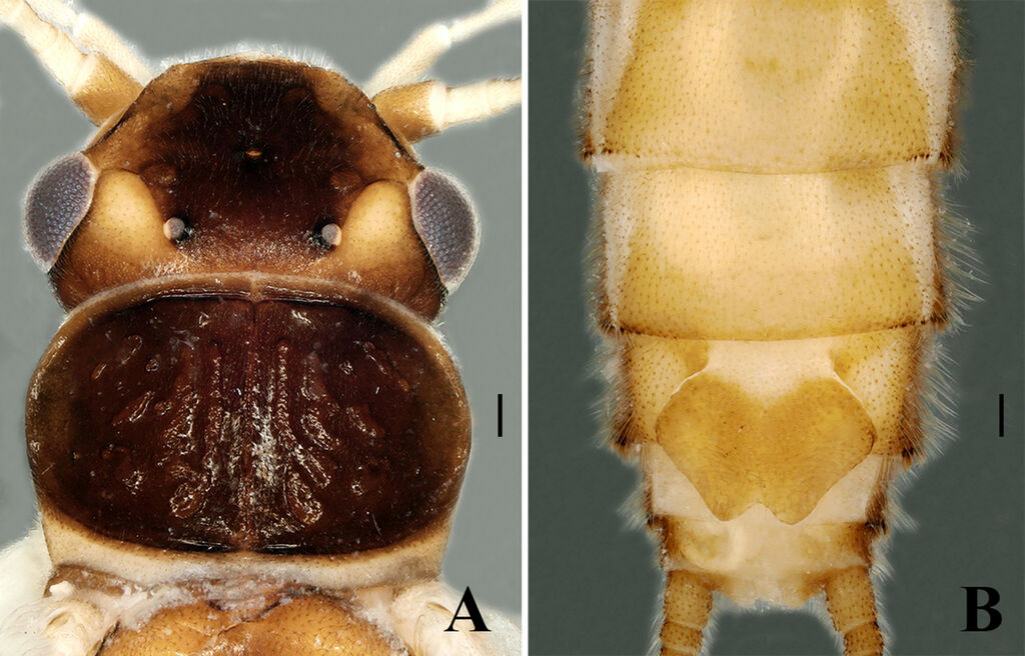
*Sweltsaligula* Rehman, Huo & Du sp. n. female paratype. **A** head and pronotum; **B** terminalia ventral view. Scale bar 0.1 mm.

Body length 9.5–10 mm (n = 3); forewing length 8.0–8.5 mm, hind-wing length 7.0–7.5 mm. Habitus is generally similar to the male. Head and pronotum are darker than the males. Abdominal tergum 1–4 dorsally with median stripe of brown trapezoidal spots and terga 5–7 with median stripe of oval shape (Fig. 2B). Sternum 8 bears a triangular sclerotised subgenital plate, reaching near to the posterior margin of sternum 9; covered with tiny hairs. Posterior margin of the subgenital plate is with a large triangular posteromedial notch (Fig. 5A–B).

##### Egg

Unknown

##### Nymph

**Figure 6.**
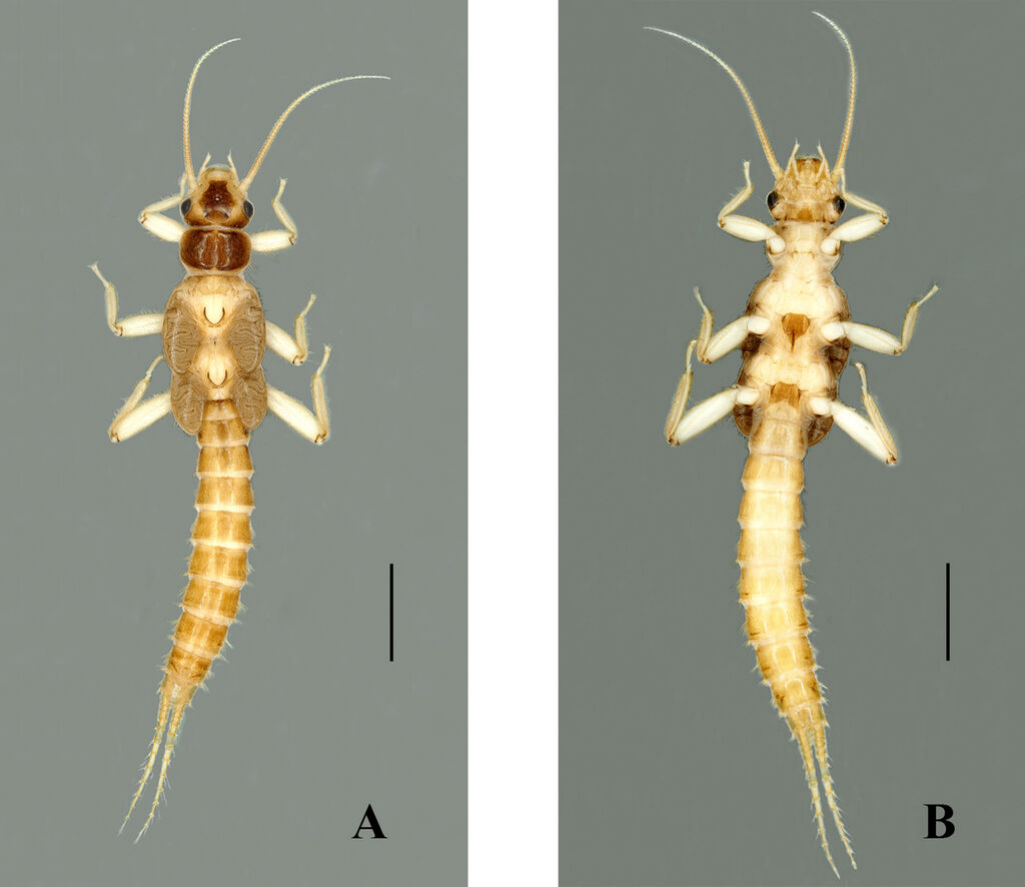
*Sweltsaligula* Rehman, Huo & Du sp. n. nymph. **A** dorsal view; **B** ventral view. Scale bar 1 mm.

**Figure 7.**
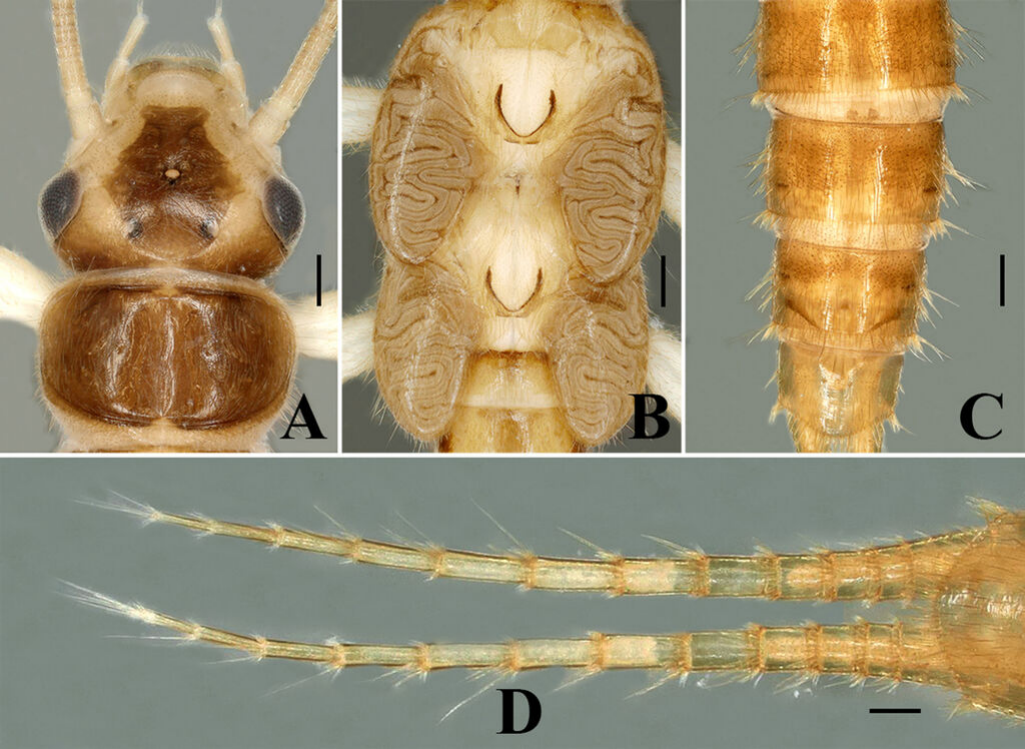
*Sweltsaligula* Rehman, Huo & Du sp. n. nymph. **A** head and pronotum; **B** thorax and wing-pads; **C** terminalia dorsal view; **D** cerci dorsal view. Scale bar 0.1 mm.

Habitus (Fig. 6A–B). Body length 7.0–7.5 mm (n = 2). General body colour brown with an obscure pattern of darker brown. Head primarily brown, ocellar area and frons bear a rectangular darker pigmentation, except for pale areas near antennal bases and along anterior margin of frons covering clypeus; dark area of head consists of several oval spots, the two pale spots on the lateral sides of median ocellus and two spots on frons above median ocellus, occiput mostly pale. Compound eyes dark, ocelli greyish, anterior ocellus paler with dark black margin. Pronotum disc dark brown with darker submarginal bands along posterior and anterior margins; the lateral margins brown, disc without an obvious pattern (Fig. 7A). The pronotal setal fringes are well-developed, with two-thirds of anterior margin and complete along posterior margin. Mesonotae and metanotae with distinct U-shaped marking, basal bristle patch of mixed length setae; wing pad developed and margins with few bristles (Fig. 7B). The entire thoracic segments and abdomen are covered with pale brown hairs. Abdominal segments 7–9 covered with small and long bristles; lateral bristle is typical. Cerci pale brown (Fig. 7C). Cersal bristles are well-developed, apical segmented whorls; basal are segmented with shorter bristles, the apical segment with at least one or two dorsal bristles equal in length or slightly longer than the following segment (Fig. 7D). Leg pale, femora and tibia covered with sparse dorsal silky setae.

#### Diagnosis

This new species is characterised by the dark pigmentation of the head, pronotum and the shape of epiproct. Tergum 9 is sclerotised without any ridge and stripe. Epiproct is long and spoon-shaped, apically rounded in dorsal view; in lateral aspect, the epiproct is thin and parallel for its most part, apically wide and slightly curved at the apex.

#### Etymology

The name of the new species refers to the shape of the epiproct that is spoon-shaped. The Latin “*ligula*” means spoon shape.

#### Distribution

China (Guizhou Province).

#### Taxon discussion

**Figure 8.**
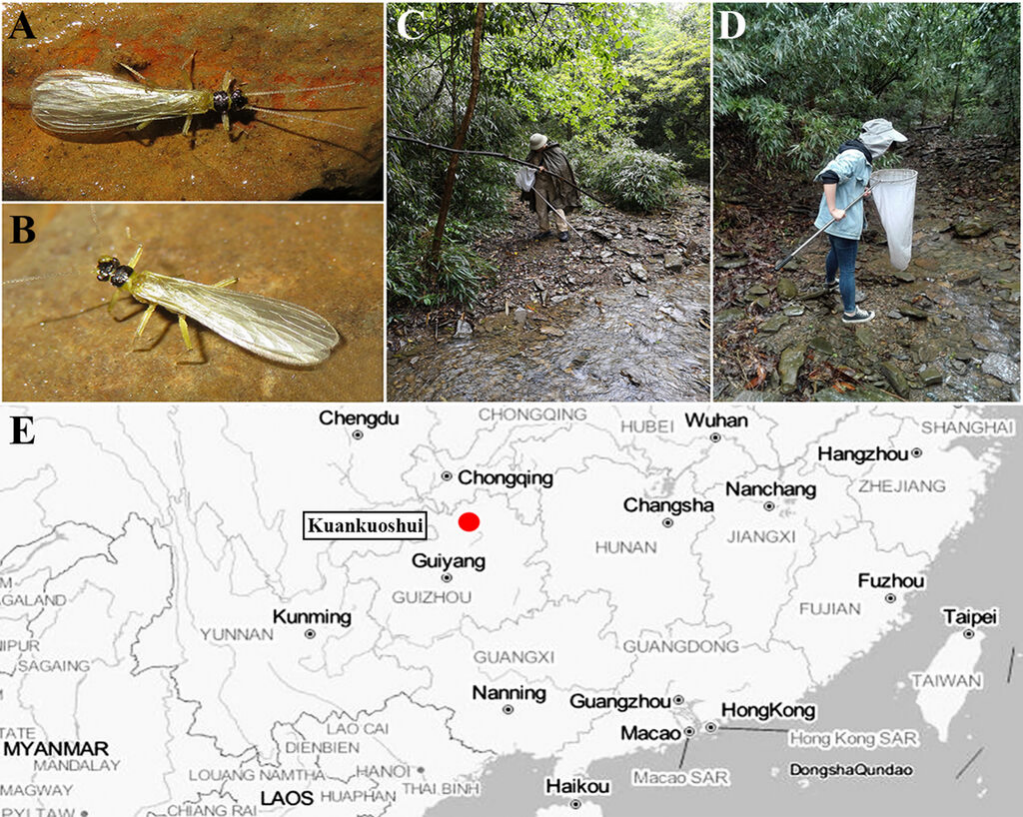
*Sweltsaligula* Rehman, Huo & Du sp. n. type locality. **A**, **B** adults on the rock; **C, D** habitat; **E** distributional map, with type locality marked in red.

The new species is very similar to *Sweltsacolorata* Zhiltzova & Levanidova, 1978 in Teslenko and Zhiltzova (2009). The new species can be distinguished from *S.colorata* by the head, pronotum pigmentation and the shape of the epiproct. The head and pronotum disc of the new species are highly sclerotised and darker than *S.colorata* (Fig. 1A). The abdominal tergum stripe of *S.colorata* is similar to the new species. The epiproct of the new species is broad with a spoon-bowl-shaped apex in dorsal view (Fig. 3A–B), while the epiproct of *S.colorata* has a pointed apex and is more slender than the new species (see figs. 505 and 506 in Teslenko and Zhiltzova 2009). In the lateral aspect, the epiproct of the new species is slender and parallel for its most part, apically wide and slightly curved, which easily distinguished the new species. The female of the new species is different from the female of *S.colorata*. The female subgenital plate of the new species posteriorly has a medial notch, while the female of *S.colorata* is without any prominent notch, characteristics which easily differentiate these two females. The nymph lives on the surface of the stream near the waterfall (stream width is 2–3 m and the water depth is no more than 30 cm). The adults often emerged under the wide rocks (Fig. 8A–B) and stayed on the surrounding trees, especially in bamboo (Fig. 8C–D).

## Supplementary Material

XML Treatment for Sweltsa ligula

## References

[B7639339] Chen Z. T., Du Y. Z. (2015). A new species of *Suwallia* (Plecoptera: Chloroperlidae) from China. Zootaxa.

[B7639348] Chen Z. T., Du Y. Z. (2016). A new species of *Haploperla* from China (Plecoptera, Chloroperlidae). ZooKeys.

[B7639357] Chen Z. T., Du Y. Z. (2016). Two new species of *Haploperla* (Plecoptera: Chloroperlidae) from China. Zootaxa.

[B7639366] Chen Z. T., Du Y. Z. (2017). A new species of *Sweltsa* (Plecoptera: Chloroperlidae) from China, with a key to the Sweltsa males of China. Zootaxa.

[B7639375] Chen Z. T. (2019). Review of the genus *Suwallia* (Plecoptera: Chloroperlidae) from China with description of Suwallia jihuae sp. nov. from Sichuan Province. Zootaxa.

[B7639565] De Figueroa J. M. Tierno, Fochetti R. (2002). *Sweltsayunnan*, sp. nov., a new stonefly from China (Plecoptera: Chloroperlidae). Oriental Insects.

[B7639410] DeWalt R. E., Maehr M. D., Hopkins H., Neu-Becker U., Stueber G. (2022). Plecoptera Species File. Version.

[B7639392] Dong W. B., Cui J. X., Li W. H. (2018). A new species of *Sweltsa* (Plecoptera: Chloroperlidae) from Sichuan Province of southwestern China. Zootaxa.

[B7639384] Du Y. Z. (1999). A taxonomic study on Plecoptera from China.

[B7816550] Illies J. (1966). Katalog der rezenten Plecoptera. Das Tierreich.

[B7639420] Li W. H., Wang R. F. (2011). A new species of *Alloperla* (Plecoptera: Chloroperlidae) from China. Zootaxa.

[B7639429] Li W. H., Yao G., Qin X. F. (2013). *Haploperlachoui* sp. n. (Plecoptera: Chloroperlidae), a remarkable new stonefly from Qinling Mountains of China.. Zootaxa.

[B7639438] Li W. H., Yang J., Yao G. (2014). Review of the genus *Sweltsa* (Plecoptera: Chloroperlidae) in China. Journal of Insect Science.

[B7639456] Li W. H., Murányi D., Shi L. (2015). The first record of genus *Suwallia* Ricker, 1943 (Plecoptera: Chloroperlidae) from China. Illiesia.

[B7639465] Li W. H., Murányi D., Shi L. (2015). New species records of *Suwallia* Ricker, 1943 (Plecoptera: Chloroperlidae) from China, with description of the nymph of *S.decolorata* Zhiltzova & Levanidova, 1978.. Zootaxa.

[B7639483] Li W. H., Pan Z. H., Liu R. J. (2017). Description of *Sweltsatibetensis* sp. n. (Plecoptera: Chloroperlidae) from Tibet Autonomous Region of China.. Zootaxa.

[B7639501] Li W. L., Wang Y. Y., Li W. H., Li M. Y. (2021). Two new species *of Sweltsa* (Plecoptera: Chloroperlidae) from China. Zootaxa.

[B7639511] Li W. L., Wang Y. Y., Wang Y., Li W. H. (2021). A new species of *Suwallia* Ricker, 1943 from Japan, and the identity of *Alloperlateleckojensis* Šámal, 1939 (Plecoptera: Chloroperlidae). Zootaxa.

[B7639520] Mo R. R., Ye J. P., Wang G. Q., Li W. H. (2020). The first record of the family Chloroperlidae (Plecoptera) from the Guangxi Zhuang Autonomous Region of southern China, with description of a new species of *Sweltsa* Ricker, 1943. Zootaxa.

[B7639538] Nelson C. H., Hanson J. F. (1968). Two new species of *Alloperla* (Plecoptera: Chloroperlidae) from China. Journal of the Kansas Entomological Society.

[B7639529] Nelson C. H., Hanson J. F. (1969). Genus *Utaperla* (Plecoptera-Chloroperlidae). Pan-Pacific Entomologist.

[B7816515] Rehman A., Huo Q. B., Du Y. Z. (2022). ﻿A new species of *Suwallia* Ricker, 1943 (Plecoptera, Chloroperlidae) from southwestern China, with an updated key to male *Suwallia* species. ZooKeys.

[B7816524] Ricker W. E. (1943). Stoneflies of southwest British Columbia.

[B7819022] Shi W. J., Wang H. L., Li W. H. (2022). A new species and three new records of Chloroperlidae (Plecoptera) from northeastern China. Zootaxa.

[B7639547] Stark B. P., Sivec I. (2009). *Sweltsawui* and *Haploperlavalentinae* (Plecoptera: Chloroperlidae), two new stoneflies from Sichuan Province, China. Illiesia.

[B7641403] Surdick R. F. (1985). Nearctic genera of Chloroperlinae (Plecoptera: Chloroperlidae). Illinois Biological Monographs 54.

[B7639583] Teslenko V. A., Zhiltzova L. A. (2009). Key to the stoneflies (Insecta, Plecoptera) of Russia and adjacent countries. Imagines and nymphs. Dalnauka Vladivostok. Dalnauka Vladivostok.

[B7639592] Wu C. F. (1938). *Plecopterorumsinensium*: A monograph of the stoneflies of China (Order Plecoptera).

[B7639600] Wu C. F. (1973). New species of Chinese stoneflies (Order Plecoptera). Acta Entomologica Sinica.

[B7639609] Yang D., Li W. H., Chen Yi-Yu (2018). Species Catalogue of China.

[B7639622] Zhiltzova L. A., Zwick P. (1971). Notes on Asiatic Chloroperlidae (Plecoptera), with descriptions of new species. Entomologisk Tidskrift.

